# Best Parameters of Heart Rate Variability for Assessing Autonomic Responses to Brief Rectal Distention in Patients with Irritable Bowel Syndrome

**DOI:** 10.3390/s23198128

**Published:** 2023-09-28

**Authors:** M. Khawar Ali, Shiyuan Gong, Borko Nojkov, Colin Burnett, Jiande D. Z. Chen

**Affiliations:** 1 Michigan Medicine, Department of Gastroenterology and Hepatology, University of Michigan, Ann Arbor, MI 48109, USAbnojkov@med.umich.edu (B.N.); bucolin@med.umich.edu (C.B.); 2 Department of Biomedical Engineering, University of Michigan, Ann Arbor, MI 48109, USA; gongsh@umich.edu

**Keywords:** HRV, IBS, rectal distension, signal processing, habituation

## Abstract

Heart rate variability (HRV) has been used to measure autonomic nervous system (ANS) activity noninvasively. The purpose of this study was to identify the most suitable HRV parameters for ANS activity in response to brief rectal distension (RD) in patients with Irritable Bowel Syndrome (IBS). IBS patients participated in a five-session study. During each visit, an ECG was recorded for 15 min for baseline values and during rectal distension. For rectal distension, a balloon was inflated in the rectum and the pressure was increased in steps of 5 mmHg for 30 s; each distension was followed by a 30 s rest period when the balloon was fully deflated (0 mmHg) until either the maximum tolerance of each patient was reached or up to 60 mmHg. The time-domain, frequency-domain and nonlinear HRV parameters were calculated to assess the ANS activity. The values of each HRV parameter were compared between baseline and RD for each of the five visits as well as for all five visits combined. The sensitivity and robustness/reproducibility of each HRV parameter were also assessed. The parameters included the Sympathetic Index (SI); Root Mean Square of Successive Differences (RMSSD); High-Frequency Power (HF); Low-Frequency Power (LF); Normalized HF Power (HFn); Normalized LF Power (LFn); LF/HF; Respiratory Sinus Arrhythmia (RSA); the Poincare Plot’s SD1, SD2 and their ratio; and the pNN50, SDSD, SDNN and SDNN Index. Data from 17 patients were analyzed and compared between baseline and FD and among five sessions. The SI was found to be the most sensitive and robust HRV parameter in detecting the ANS response to RD. Out of nine parasympathetic parameters, only the SDNN and SDNN Index were sensitive enough to detect the parasympathetic modulation to RD during the first visit. The frequency-domain parameters did not show any change in response to RD. It was also observed that the repetitive RD in IBS patients resulted in a decreased autonomic response due to habituation because the amount of change in the HRV parameters was the highest during the first visit but diminished during subsequent visits. In conclusion, the SI and SDNN/SDNN Index are most sensitive at assessing the autonomic response to rectal distention. The autonomic response to rectal distention diminishes in repetitive sessions, demonstrating the necessity of randomization for repetitive tests.

## 1. Introduction

Even though the gastrointestinal (GI) tract is innervated by the enteric nervous system (ENS), which provides significant autonomy to the GI functions, the supervisory input from the central nervous system (CNS) is supplied by autonomic innervations of the GI tract, which communicate with the ENS to modulate GI functions. The autonomic nervous system innervation to the GI tract includes parasympathetic and sympathetic innervations. The parasympathetic innervations to the upper GI tract are supplied by the vagus nerve and innervations of the lower GI tract are supplied by the sacral parasympathetic nerves. The sympathetic innervations are supplied by sympathetic nerves originating from the thoracic and lumber spinal cord. Dysfunction in autonomic activity is one of the major causes of functional GI disorders including Irritable Bowel Syndrome (IBS) [1]. IBS is an extremely common GI disorder with no identifiable structural or biochemical impairment in affected patients. Clinically, IBS is characterized by abdominal pain and an associated change in bowel movement frequency or consistency [2,3,4].

One of the common features of IBS is visceral hypersensitivity, which is defined as a reduction in pain and discomfort thresholds. Although the mechanism of triggering visceral hypersensitivity remains unclear, it is believed to be associated with the modulation of visceral sensory neurons [5,6]. Visceral hypersensitivity is common in IBS patients, and it has been used as a biomarker of the disease [7]. Several studies have reported lowered sensory thresholds in response to rectal balloon distensions in IBS patients [8,9]. Rectal distension is known to modulate autonomic nervous system activity [10,11,12,13] and can be used to study the pathophysiology of IBS [14]. This is achieved by inflating a balloon in the rectum at various pressures to induce the sensation of pain and discomfort, which triggers the autonomic response. Comparing the autonomic response to rectal distension between IBS patients and healthy controls allows for the identification of impaired autonomic function that has been implicated in the development of IBS [6].

Heart rate variability (HRV) is a noninvasive method used to assess autonomic nervous system activity. HRV has been used to study the autonomic activity associated with normal GI functions, autonomic dysfunction relevant to the pathophysiology of certain GI disorders and to assess therapeutic effectiveness [1,3]. Several HRV parameters have been developed and used for the assessment of parasympathetic and sympathetic activities. However, we have previously shown that the capability of HRV parameters to measure autonomic activity is influenced by various conditions and tests [3,15]. Therefore, there is a need to identify the most appropriate HRV parameters to assess autonomic function during rectal distension.

The aim of this study was to determine the most sensitive HRV parameters for the assessment of autonomic function in response to brief rectal distension. We tested several HRV parameters (the time domain, frequency domain and nonlinear domain) used for parasympathetic/sympathetic activities and autonomic balance assessment in association with rectal distension in patients with IBS. The change in the modulation degree for each parameter with repeated rectal distensions within the same group of patients and the correlation between the HRV parameters and pain scores during rectal distension were studied.

## 2. Methods

### 2.1. Selection Criteria for the Patients

Nineteen patients that fulfilled the Rome 4 diagnostic criteria for Irritable Bowel Syndrome with constipation (IBS-C) were recruited and completed the study protocol. To be selected for this study, patients had to be male or female above the age of 18 and willing to adhere to all procedures throughout the study. All participants had chronic abdominal pain of at least 3 on a 10-point pain-severity rating scale that was assessed at baseline. The study exclusion criteria were: (i) the presence of an alternate structural disorder that can explain abdominal pain; (ii) prior abdominal or anorectal surgery; (iii) the use of anticoagulant/antiplatelet medications, opioids or other pain-relief medications; (iv) pregnancy or breastfeeding; and (v) a history of allergic reactions to components of ECG electrodes.

### 2.2. Study Design

All the participants signed an informed consent document upon study enrollment. This study entailed five different sessions performed on separate days when each participant underwent a standardized rectal balloon distension protocol in order to assess the pain perception and rectal sensation. An electrocardiogram (ECG) was recorded for 15 min at baseline and during the rectal distention period for each individual to assess the autonomic function. Prior to each of these visits, the participants were fasting for 12 h and were required to not take any medications known to affect gastrointestinal pain perception for 48 h. Each individual study was performed in a private room within the Gastrointestinal Physiology Laboratory at the University of Michigan.

Three ECG electrodes were placed on the participants to record the ECG signal. One electrode was placed at the apex area of the heart, one was placed at the right edge of the sternum and one reference electrode was positioned at the right side of the chest. The skin area where the electrodes were to be placed was cleaned by using skin prep materials before the placement.

For rectal distension, a barostat device (manufactured by G&J Electronics Inc, Toronto, ON, Canada) was used. This FDA-approved device allows for the maintenance of the prespecified balloon pressure and the delivery of the controlled distension of the rectum. The barostat was connected to a thin, flexible, polyvinyl catheter with a polyethylene balloon at the tip (a 500 mL capacity and 10 cm long, Mui Scientific, Mississauga, ON, Canada). This balloon was positioned 5–15 cm into the study participant’s rectum.

After the initial 15 min baseline ECG recording and the insertion of the barostat catheter, rectal distention was performed at different pressures from 5 mmHg to the maximum tolerable pressure with an increment of 5mmHg. Each distension lasted for 30 s and was followed by a 30 s rest period when the balloon was deflated. Each participant was asked to score the RD-induced pain on a 10-point visual analog scale at each distension pressure point.

There was at least a 4-day interval between any 2 consecutive sessions for each participant.

### 2.3. Heart Rate Variability (HRV) Parameters

The interbeat interval (RR interval) signal was calculated from the recorded ECG signal to calculate the HRV parameters (shown in Table 1); the Sympathetic Index (SI), Root Mean Square of Successive Differences (RMSSD), Respiratory Sinus Arrythmia (RSA) and SD1 and SD2 of the Poincare Plot and their ratio (SD2/SD1) were calculated by using MATLAB code developed by the authors. The frequency-domain parameters of the SDSD, SDNN and SDNN Index and HF, LF, HFn, LFn and LF/HF were calculated by using previously validated software developed in the lab.

The robustness/reproducibility of each HRV parameter was evaluated by comparing their values among each visit as described below.

### 2.4. Statistical Analysis

A Shapiro–Wilk normality test was performed on each HRV parameter before each comparison. If the parameter was normally distributed, the paired *t*-test was applied to investigate the difference between the baseline and rectal distension. Otherwise, if not normally distributed, a Wilcoxon test was performed to investigate the differences.

To test the reproducibility of the HRV parameters, the baseline and RD values of each visit were compared. The baseline values of all the five visits were compared by using a one-way ANOVA for the normally distributed data and a Kruskal–Wallis test for the data that were not normally distributed, followed by a Dunn’s multiple comparison test. The procedure was repeated by using HRV values during rectal distension and compared among 5 visits.

## 3. Results

**Figure 1:**
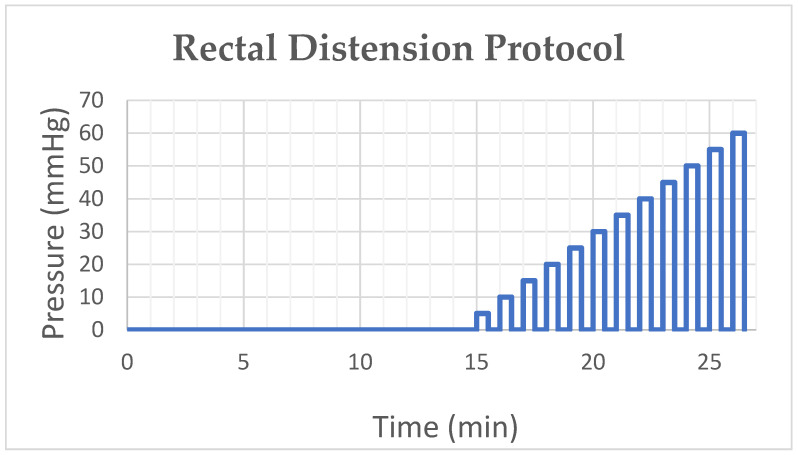
Rectal distension protocol: (i) 15 min of baseline HRV recording after placing the balloon inside the rectum; (ii) the distension pressure was increased in steps of 5 mmHg for 30 s followed by a rest period (0 mmHg) of 30 s after each distension step, up to 60 mmHg or maximum tolerance level of the patient.

Out of the 19 patients, 2 patients were excluded from the data analysis because the HRV data from these patients were considered outliers. For example, the average baseline SI value for five visits for one of these two patients was 355.82 s^−2^ vs. the average baseline SI value of 36.12 s^−2^ for all five visits (Table 9). The second patient had a baseline SI value of 294.82 vs. the average baseline SI value of 36.26 during the second visit, and during the fifth visit, the baseline SI value was 271.95 vs. the average value of 38.72 s^−2^; additionally, their ECG during rectal distension was also not recorded due to technical difficulties (Figure 1).

All the patients completed the study protocol (finished all five sessions and completed the RD protocol). The mean maximal tolerable RD pressure was 46.48 ± 12.60 mmHg, and the average time of rectal distension was 9.21 ± 2.38 min.

In Table 2, Table 3, Table 4, Table 5 and Table 6, the HRV parameters are compared between baseline and RD for each visit. The overall trend was an increase in the sympathetic and a decrease in the parasympathetic activity and a shift of autonomic balance toward the sympathetic nervous system during RD compared to the baseline. However, some HRV parameters reached statistical significance while others did not, and the amount of RD-induced change was not the same in the HRV parameters, as indicated by the percentage change during each visit.

During visit 1 (Table 2), 5 out of 15 parameters showed a significant change in response to RD: a significant increase in sympathetic activity as indicated by the SI value (increased by 80.98%, *p* = 0.0131) and a significant decrease in parasympathetic activity as indicated by the SDNN (decreased by 19.58%, *p* = 0.0042) and SDNN Index (decreased by 14.94%, *p* = 0.0392), while all the other parasympathetic parameters decreased but the change was not statistically significant. SD2, which is influenced by both sympathetic and parasympathetic activity, was significantly decreased by 21.67% (*p* = 0.0014) and SD2/SD1 decreased by 22.23% (*p* = 0.0022). Accordingly, based on the data collected from visit 1, the SI value showed the largest percent increase, indicating the highest sensitivity amongst all the parameters.

During the second visit (Table 3), the amount of increase in sympathetic activity assessed by the SI was reduced to 43.51%; however, the change was still significant statistically (*p* = 0.0174). Similarly, the amount of decrease in parasympathetic activity assessed by the SDNN and SDNN Index during rectal distension was also reduced compared to that of visit 1; however, no parasympathetic parameter reached statistical significance. SD2 and SD2/SD1 decreased significantly during RD compared to baseline in visit 2 as well.

During the third (Table 4), fourth (Table 5) and fifth (Table 6) visits, no HRV parameter for sympathetic activity nor parasympathetic activity was changed significantly even though the mean values of the SI increased while most of the parasympathetic parameters decreased during RD; the amount of RD-induced change was less compared to visit 1 and 2.

As we move from visit 1 to visit 5, the amount of increase in sympathetic activity and amount of decrease in parasympathetic activity diminished after each visit. Rectal distension influenced the sympathetic nervous system more (due to the amount of change in the SI) as compared to the parasympathetic nervous system.

The frequency-domain parameters HF, LF, HFn, LFn and their ratio did not show any notable changes, possibly attributed to the short duration of the distention period (9.21 ± 2.38 min). The nonlinear parameter SD1 was consistent with the decrease in parasympathetic activity due to rectal distension, but SD2 and the ratio SD2/SD1 were not consistent with the decreased parasympathetic activity and shifting of the autonomic balance toward the sympathetic nervous system.

Upon analyzing the data by averaging the data from five visits for each patient and comparing between the baseline and rectal distention (Table 7), there was still an increase in the sympathetic system as indicated by the SI. The significant decrease in the parasympathetic system was observed in the SDNN. For the parasympathetic/sympathetic parameters, a significant decrease in SD2 was observed, while for the autonomic balance parameter, only SD2/SD1 showed a significant decrease, which meant there was a shift toward the sympathetic system.

### The Reproducibility, Sensitivity and Robustness of the HRV Parameters

The sensitivity and robustness of the HRV parameters were also the subject of interest. The standard deviations of the mean of each parameter were calculated for each visit to identify the variability. As shown in Table 8 and Table 9, the averages for each parameter were consistent across visits as the maximum and the minimum of each parameter could be encompassed within two standard deviations from the mean. The baseline and RD values of the HRV parameters were compared among all five visits to test the robustness. No significant change was observed among the baseline values of any HRV parameter during any visit, and same was the case for the HRV values during rectal distension.

## 4. Discussions

In this study, we aimed to identify the most reliable, sensitive and reproducible/robust HRV parameters associated with the autonomic responses to rectal distension in IBS patients. We also aimed to identify the change in autonomic response during the repeated sessions of rectal distension. Since each patient had five visits separated by a minimum of 4 days, the comparison of HRV parameters between the baseline and rectal distension was carried out separately for each visit to observe the altered autonomic response due to the repetitive rectal distension by combining all the visits as well by averaging all five visits of each patient and comparing these values for all the patients. The results indicated that the autonomic response to rectal distension was the highest during the first visit and diminished during subsequent visits. In the first visit, the average value of the SI changed from 29.12 to 52.69, which is an increase of 80.98% with a *p*-value of 0.0131. This increase in the SI was 43.51% (*p* = 0.0174) in the second visit; 36.02% (*p* = 0.0946, not statistically significant) in the third visit; and 32.64% and 40.95% in 4th and 5th visits, respectively, with no statistical significance. Similarly, out of nine parasympathetic HRV parameters used, only the SDNN and SDNN Index showed a significant decrease in their average values during visit 1 only. On average, the value of the SDNN decreased by 19.58% (*p* = 0.0042) and the SDNN Index decreased by 14.94% (*p* = 0.0392) with no change in all the following visits. No other parasympathetic parameter showed any significant change in any visit. The RMSSD and SD1 also showed a slight decrease in average values during rectal distension during the first visit only, but these changes were not statistically significant.

The frequency-domain parameters HF, HFn, RSA, LF, LFn and LF/HF and their ratio did not show any change during any visit, which could be due to the fact that the duration of the HRV recording during rectal distension was relatively brief (an average of 9.2 min). The overall comparisons also indicated similar results; i.e., a significant increase in sympathetic and a decrease in parasympathetic nervous system activity. During the first visit, the difference between the average baseline values of all the HRV parameters from their respective mean (calculated from the average values of each visit) was the highest, which diminished in subsequent visits.

This indicates a diminishing autonomic response to the rectal distension over subsequent sessions, which can be explained by the concept of habituation, a decrease in responsiveness to a repeated stimulus caused by nonassociative learning [16]. A major consequence of a repetitive stimulus is the decrease in the frequency or amplitude of the response. A habituation or learning phenomenon is known to have occurred when the decrement is not caused by the physiological changes at the sensory or motor levels [17,18]. Therefore, it is crucial to take into account the diminished autonomic response due to habituation when investigating the autonomic response to any repetitive stimulus, including rectal distension. It is very important to randomize the trails during the study design.

We attempted to correlate the HRV parameters with the pain scores to identify whether any of these HRV parameters could be used as biomarkers of the pain. In addition, we were also curious whether these parameters during baseline could be used as predictors for the pain score during rectal distension. Adding pain scores across visits showed a correlation with some HRV parameters. Only the frequency-domain parameters showed a correlation with the pain score. However, because the frequency-domain parameters were not sensitive enough to detect the autonomic modulation due to rectal distension, the significant correlations of these parameters were not trustworthy.

The SI was the only sympathetic parameter used in our study; the average value of the SI for visit 2–5 was within one SD of the mean while this first one was within two SD. It is found to be a sensitive as well as robust HRV parameter as it only varied by less than 10% during the baseline of each visit, and it was sensitive enough to record the sympathetic activation during rectal distension (visit 1 and 2). Among the time-domain parasympathetic parameters, the RMSSD showed the smallest variation (nonsignificant) due to rectal distension, and among the frequency-domain parasympathetic HRV parameters, HFn showed the smallest variation from its mean; the only nonlinear SD1 (Poincare Plot) changed by only 4.28% on average from its mean of all the visits. The results of the frequency-domain parameters indicated that even if they are robust, they did not show any change due to rectal distension, which indicated that they are not sensitive enough if the recording duration is brief (in this study, the duration was 9.21 ± 2.38 min). The SDNN (*p* = 0.0042 during visit 1) and SDNN Index (*p* = 0.0392 during visit 1) were proved to be the sensitive parasympathetic parameters during rectal distension. The SNDD Index varied only 5.68% from its baseline mean from all visits whereas the SNDD varied by 8.56% when compared between baseline and rectal distension. Hence, the SI should be used for the assessment of the sympathetic response, and the SDNN/SNDD Index should be used for the assessment of the parasympathetic response to the rectal distension.

While our study comprehensively assessed various parameters of HRV, it is important to acknowledge its limitations. A primary limitation was the small sample size of 17 participants (5 visits per patient, a total of 85 visits), primarily due to the requirement for highly trained personnel to conduct the research. In future studies, expanding the participant pool will enhance the validity and reliability of our findings. Additionally, incorporating a control group consisting of healthy volunteers would provide valuable comparative insights. Therefore, a key focus for our future research endeavors will involve recruiting a larger and more diverse cohort, including healthy individuals.

In conclusion, the SI and SDNN/SDNN Index are the most sensitive HRV parameters at assessing the autonomic response to rectal distention in patients with IBS, whereas frequency-domain HRV parameters are not sensitive at assessing autonomic responses to rectal distention, probably attributed to the brief duration. These findings suggest that different HRV parameters may have to be used for specific applications. The autonomic response to rectal distention diminishes for repetitive sessions, demonstrating the importance of randomization for repetitive tests.

## Figures and Tables

**Table 1:** HRV parameters used for autonomic assessment.

**Sympathetic**	Sympathetic Index (SI) *	$\begin{array}{c} SI=\frac{AM_{o}*100\%}{2M_{o}*M_{x}DM_{n}}\\ AM_{o}=amplitude\;of\;of\;mode\\ M_{o}=mode\;of\;RR\;interval\\ M_{x}DM_{n}=range\;of\;RR\;interval \end{array}$
**Parasympathetic**	Root Mean Square (rms) of the successive differences between the RR intervals (RMSSD) *	$\sqrt{\frac{1}{n-1}\sum_{i=1}^{n-1}(RR_{i+1}-RR_{i}{)}^{2}}$ $n=total\;RR\;intervals\;RR_{i}=current\;RR\;interval\;RR_{i+1}=next\;RR\;interval$
	SDSD *	Standard deviation of successive differences (unit: ms).
	pNN50 (%) *	Percentage of successive RR intervals which differ by more than 50 ms.
	Standard deviation of NN (SDNN) *	NN: RR intervals after removing artifacts from the original ECG tracing (unit: ms).
	SDNN Index *	Average standard deviation of the NN interval in all of the 5 min segments of a 24 h recording (unit: ms).
	High Frequency (HF) ^#^	0.15~0.4 Hz (associated with 9~24 breath/min).
	Normalized High Frequency (HFn) ^#^	$nHF=\frac{HF}{LF+HF}$
	Respiratory Sinus Arrythmia (RSA) ^#^	The amplitude of the breath-induced sinusoidal oscillation of the heart rate interval.
	SD1 ˆ	$\sqrt{\frac{1}{N-1}\sum_{i=1}^{N-1}(\frac{RR_{i+1}-RR_{i}}{\sqrt{2}}{)}^{2}}$ $RR_{i}=current\;RR\;interval\;RR_{i+1}=next\;RR\;interval$
**Para/Symp**	Low Frequency (LF) ^#^	0.04~0.15 Hz (associated with 2.4~9 breath/min).
	Normalized Low Frequency (LFn) ^#^	$nLF=\frac{LF}{LF+HF}$
	SD2 ˆ	$\sqrt{\frac{1}{N-1}\sum_{i=1}^{N-1}(\frac{RR_{i}+RR_{i+1}-2\bar{RR}}{\sqrt{2}}{)}^{2}}$ $RR_{i}=current\;RR\;interval\;RR_{i+1}=next\;RR\;interval\;\bar{RR}=average\;of\;the\;two\;intevals$
**Autonomic Balance**	LF/HF ^#^	Ratio of Low Frequency to High Frequency.
	SD2/SD1 ˆ	Ratio of the lengths of major and minor axes of the Poincare Plot of RR intervals.

*: time-domain parameter; #: frequency-domain parameter; ˆ: nonlinear parameter.

**Table 2:** Comparison of HRV parameters during baseline and rectal distension for visit 1.

Visit 1 (n = 17)		Baseline (Mean ± SD)	RD (Mean ± SD)	% Change	*p*-Value
**Sympathetic**	SI	29.12 ± 14.37	52.69 ± 47.88	80.98%	0.0131
**Parasympathetic**	RMSSD	66.76 ± 30.53	62.00 ± 22.10	−7.13%	0.3147
	SDSD	71.13 ± 41.87	62.88 ± 31.85	−11.59%	0.1796
	PNN50	30.76 ± 18.18	24.75 ± 15.25	−19.55%	0.1395
	SDNN	90.33 ± 29.55	72.65 ± 25.34	−19.58%	0.0042
	SDNN Index	81.62 ± 30.85	69.43 ± 23.39	−14.94%	0.0392
	HF	45.85 ± 4.17	44.52 ± 3.53	−2.90%	0.1055
	HFn	0.49 ± 0.02	0.49 ± 0.02	−0.42%	0.37
	RSA	7.23 ± 0.80	7.00 ± 0.88	−3.09%	0.3781
	SD1	46.88 ± 21.67	43.87 ± 15.63	−6.41%	0.3731
**Para/Symp**	LF	48.16 ± 3.39	47.22 ± 3.63	−1.94%	0.3568
	LFn	0.51 ± 0.02	0.51 ± 0.02	0.40%	0.37
	SD2	112.05 ± 28.73	87.77 ± 26.21	−21.67%	0.0014
**Autonomic Balance**	LF/HF	1.05 ± 0.07	1.06 ± 0.06	0.82%	0.4338
	SD2/SD1	2.72 ± 0.94	2.12 ± 0.59	−22.23%	0.0022

**Table 3:** Comparison of HRV parameters during baseline and rectal distension for visit 2.

Visit 2 (n = 17)	HRV Parameter	Baseline (Mean ± SD)	RD (Mean ± SD)	% Change	*p*-Value
**Sympathetic**	SI	36.26 ± 14.85	52.04 ± 28.25	43.51%	0.0174
**Parasympathetic**	RMSSD	57.88 ± 23.74	56.93 ± 17.48	−1.66%	0.8679
	SDSD	52.62 ± 23.38	54.17 ± 25.01	2.94%	0.8051
	PNN50	27.25 ± 15.63	24.50 ± 13.79	−10.09%	0.2841
	SDNN	67.52 ± 18.19	66.33 ± 19.44	−1.76%	0.0693
	SDNN Index	67.52 ± 18.50	66.33 ± 21.70	−1.76%	0.554
	HF	43.21 ± 4.90	43.23 ± 3.67	0.04%	0.9878
	HFn	0.48 ± 0.03	0.49 ± 0.02	1.10%	0.399
	RSA	6.82 ± 0.57	6.66 ± 0.92	−2.34%	0.4929
	SD1	41.06 ± 16.70	40.29 ± 12.35	−1.88%	0.8484
**Para/Symp**	LF	46.52 ± 1.99	45.80 ± 3.53	−1.54%	0.2581
	LFn	0.52 ± 0.03	0.51 ± 0.02	−1.02%	0.399
	SD2	96.38 ± 22.84	82.18 ± 23.04	−14.73%	0.0298
**Autonomic Balance**	LF/HF	1.09 ± 0.12	1.06 ± 0.06	−2.51%	0.4866
	SD2/SD1	2.59 ± 0.79	2.13 ± 0.58	−17.44%	0.0445

**Table 4:** Comparison of HRV parameters during baseline and rectal distension for visit 3.

Visit 3 (n = 17)	HRV Parameter	Baseline (Mean ± SD)	RD (Mean ± SD)	% Change	*p*-Value
**Sympathetic**	SI	39.22 ± 21.38	53.35 ± 35.34	36.02%	0.0946
**Parasympathetic**	RMSSD	58.62 ± 34.00	63.68 ± 24.93	8.63%	0.1205
	SDSD	56.68 ± 34.73	59.96 ± 27.48	5.79%	0.4212
	PNN50	27.06 ± 18.38	30.04 ± 18.38	11.02%	0.7025
	SDNN	73.69 ± 24.87	71.32 ± 21.06	−3.22%	0.6387
	SDNN Index	69.25 ± 25.14	68.67 ± 19.86	−0.84%	0.8904
	HF	43.99 ± 3.94	44.37 ± 3.60	0.87%	0.8004
	HFn	0.49 ± 0.02	0.49 ± 0.02	0.60%	0.0717
	RSA	6.64 ± 0.76	6.86 ± 0.90	3.30%	0.4369
	SD1	41.41 ± 24.06	45.12 ± 17.66	8.98%	0.107
**Para/Symp**	LF	46.45 ± 2.51	46.45 ± 3.82	−0.01%	0.9341
	LFn	0.51 ± 0.02	0.51 ± 0.02	−0.57%	0.0717
	SD2	95.34 ± 30.04	88.61 ± 25.73	−7.06%	0.2034
**Autonomic Balance**	LF/HF	1.06 ± 0.07	1.04 ± 0.07	−1.53%	0.1078
	SD2/SD1	2.54 ± 0.64	2.10 ± 0.56	−17.46%	0.0044

**Table 5:** Comparison of HRV parameters during baseline and rectal distension for visit 4.

Visit 4 (n = 17)	HRV Parameter	Baseline (Mean ± SD)	RD (Mean ± SD)	% Change	*p*-Value
**Sympathetic**	SI	37.27 ± 23.47	49.43 ± 30.44	32.64%	0.2744
**Parasympathetic**	RMSSD	60.09 ± 27.71	55.20 ± 19.48	−8.13%	0.5043
	SDSD	60.47 ± 30.25	53.08 ± 23.96	−12.22%	0.3894
	PNN50	28.56 ± 18.61	20.94 ± 13.23	−26.70%	0.1188
	SDNN	74.23 ± 22.44	70.04 ± 23.67	−5.65%	0.9612
	SDNN Index	69.40 ± 22.75	71.86 ± 24.74	3.56%	0.4117
	HF	44.38 ± 4.74	43.26 ± 3.61	−2.51%	0.4457
	HFn	0.49 ± 0.02	0.48 ± 0.02	−1.80%	0.151
	RSA	6.76 ± 0.77	6.98 ± 0.69	3.28%	0.0965
	SD1	41.78 ± 19.77	39.10 ± 13.79	−6.42%	0.9101
**Para/Symp**	LF	46.12 ± 2.99	46.70 ± 3.23	1.26%	0.1236
	LFn	0.51 ± 0.02	0.52 ± 0.02	1.73%	0.151
	SD2	93.34 ± 28.02	91.44 ± 31.50	−2.03%	0.9753
**Autonomic Balance**	LF/HF	1.05 ± 0.08	1.08 ± 0.07	3.48%	0.0959
	SD2/SD1	2.49 ± 0.81	2.41 ± 0.86	−3.38%	0.6391

**Table 6:** Comparison of HRV parameters during baseline and rectal distension for visit 5.

Visit 5 (n = 17)	HRV Parameter	Baseline (Mean ± SD)	RD (Mean ± SD)	% Change	*p*-Value
**Sympathetic**	SI	38.72 ± 30.54	54.57 ± 54.02	40.95%	0.0674
**Parasympathetic**	RMSSD	57.64 ± 23.16	58.38 ± 22.16	1.29%	0.3475
	SDSD	57.92 ± 23.40	55.60 ± 25.64	−4.01%	0.9539
	PNN50	30.65 ± 17.59	25.44 ± 18.36	−17.00%	0.0974
	SDNN	80.89 ± 25.72	69.08 ± 23.36	−14.60%	0.1209
	SDNN Index	72.90 ± 21.73	69.05 ± 25.92	−5.29%	0.8407
	HF	43.85 ± 3.88	43.76 ± 3.97	−0.21%	0.6671
	HFn	0.48 ± 0.02	0.48 ± 0.02	−0.16%	0.8066
	RSA	6.90 ± 0.63	6.81 ± 1.43	−1.23%	0.5282
	SD1	40.62 ± 16.14	41.33 ± 15.67	1.73%	0.2891
**Para/Symp**	LF	46.87 ± 2.57	46.98 ± 3.63	0.24%	0.6982
	LFn	0.52 ± 0.02	0.52 ± 0.02	0.15%	0.8066
	SD2	106.57 ± 34.63	89.14 ± 29.30	−16.36%	0.1181
**Autonomic Balance**	LF/HF	1.07 ± 0.07	1.08 ± 0.08	0.37%	0.9294
	SD2/SD1	2.84 ± 0.88	2.28 ± 0.57	−19.96%	0.0207

**Table 7:** Comparison of HRV parameters during baseline and rectal distension averaging all five visits for each patient.

Average of Visits (n = 17)	HRV Parameter	Baseline (Mean ± SD)	RD (Mean ± SD)	% Change	*p*-Value
**Sympathetic**	SI	36.10 ± 14.79	53.15 ± 28.46	47.24%	0.0013
**Parasympathetic**	RMSSD	61.06 ± 24.17	59.63 ± 16.61	−2.33%	0.7467
	SDSD	60.60 ± 24.56	57.01 ± 21.22	−5.92%	0.4379
	PNN50	29.31 ± 15.52	25.15 ± 13.55	−14.18%	0.067
	SDNN	79.60 ± 19.48	69.66 ± 18.76	−12.48%	0.0198
	SDNN Index	73.11 ± 19.98	69.11 ± 19.48	−5.48%	0.3468
	HF	44.27 ± 3.65	43.85 ± 2.95	−0.95%	0.4021
	HFn	0.39 ± 0.13	0.35 ± 0.13	−8.25%	0.1106
	RSA	6.87 ± 0.58	6.90 ± 0.75	0.39%	0.4514
	SD1	42.94 ± 17.26	42.22 ± 11.76	−1.68%	0.5791
**Para/Symp**	LF	46.81 ± 2.25	46.54 ± 2.98	−0.57%	0.6868
	LFn	0.61 ± 0.13	0.64 ± 0.13	5.12%	0.0941
	SD2	102.19 ± 23.15	88.17 ± 22.31	−13.72%	0.0093
**Autonomic Balance**	LF/HF	1.06 ± 0.07	1.06 ± 0.06	0.03%	0.8153
	SD2/SD1	2.65 ± 0.64	2.21 ± 0.44	−16.69%	0.0032

**Table 8:** Sensitivity and robustness of HRV parameters during baseline among all 5 visits.

Baseline															
	Symp.	Parasympathetic									Para/Symp.			Autonomic Balance	
	SI	RMSSD	SDSD	PNN50	SDNN	SDNN Index	HF	HFn	RSA	SD1	LF	LFn	SD2	LF/HF	SD2/SD1
Visit 1	29.12	66.76	71.13	30.76	90.33	81.62	45.85	0.49	7.23	46.88	48.16	0.51	112.05	1.05	2.72
Visit 2	36.26	57.88	52.62	27.25	67.52	67.52	43.21	0.48	6.82	41.06	46.52	0.52	96.38	1.09	2.59
Visit 3	39.22	58.62	56.68	27.06	73.69	69.25	43.99	0.49	6.64	41.41	46.45	0.51	95.34	1.06	2.54
Visit 4	37.27	60.09	60.47	28.56	74.23	69.40	44.38	0.49	6.76	41.78	46.12	0.51	93.34	1.05	2.49
Visit 5	38.72	57.64	57.92	30.65	80.89	72.90	43.85	0.48	6.90	40.62	46.87	0.52	106.57	1.07	2.84
Mean	36.12	60.20	59.77	28.86	77.33	72.14	44.25	0.49	6.87	42.35	46.82	0.51	100.73	1.06	2.64
SD	4.08	3.79	6.96	1.79	8.67	5.65	0.99	0.00	0.22	2.57	0.79	0.00	8.14	0.02	0.14

**Table 9:** Sensitivity and robustness of HRV parameters during rectal distension among all 5 visits.

	Rectal Distension														
	Symp.	Parasympathetic									Para/Symp			Autonomic Balance	
	SI	RMSSD	SDSD	PNN50	SDNN	SDNN Index	HF	HFn	RSA	SD1	LF	LFn	SD2	LF/HF	SD2/SD2
Visit 1	52.69	62.00	62.88	24.75	72.65	69.43	44.52	0.49	7.00	43.87	47.22	0.51	87.77	1.06	2.12
Visit 2	52.04	56.93	54.17	24.50	66.33	66.33	43.23	0.49	6.66	40.29	45.80	0.51	82.18	1.06	2.13
Visit 3	53.35	63.68	59.96	30.04	71.32	68.67	44.37	0.49	6.86	45.12	46.45	0.51	88.61	1.04	2.10
Visit 4	49.43	55.20	53.08	20.94	70.04	71.86	43.26	0.48	6.98	39.10	46.70	0.52	91.44	1.08	2.41
Visit 5	54.57	58.38	55.60	25.44	69.08	69.05	43.76	0.48	6.81	41.33	46.98	0.52	89.14	1.08	2.28
Mean	52.42	59.24	57.14	25.13	69.88	69.07	43.83	0.48	6.86	41.94	46.63	0.52	87.82	1.07	2.21
SD	1.91	3.53	4.14	3.25	2.40	1.98	0.60	0.00	0.14	2.50	0.55	0.00	3.44	0.02	0.13

## Data Availability

The data will be made available upon request.
